# Characterizing Continuous Manipulation Families for Dexterous Soft Robot Hands

**DOI:** 10.3389/frobt.2021.645290

**Published:** 2021-04-13

**Authors:** Jiatian Sun, Jonathan P. King, Nancy S. Pollard

**Affiliations:** Foam Robotics Lab, The Robotics Institute, Carnegie Mellon University, Pittsburgh, PA, United States

**Keywords:** robot hand, manipulation, dexterity, manipulation planning, robot hand design, Gaussian Mixture Model, soft robot, manipulation families

## Abstract

There has been an explosion of ideas in soft robotics over the past decade, resulting in unprecedented opportunities for end effector design. Soft robot hands offer benefits of low-cost, compliance, and customized design, with the promise of dexterity and robustness. The space of opportunities is vast and exciting. However, new tools are needed to understand the capabilities of such manipulators and to facilitate manipulation planning with soft manipulators that exhibit free-form deformations. To address this challenge, we introduce a sampling based approach to discover and model continuous families of manipulations for soft robot hands. We give an overview of the soft foam robots in production in our lab and describe novel algorithms developed to characterize manipulation families for such robots. Our approach consists of sampling a space of manipulation actions, constructing Gaussian Mixture Model representations covering successful regions, and refining the results to create continuous successful regions representing the manipulation family. The space of manipulation actions is very high dimensional; we consider models with and without dimensionality reduction and provide a rigorous approach to compare models across different dimensions by comparing coverage of an unbiased test dataset in the full dimensional parameter space. Results show that some dimensionality reduction is typically useful in populating the models, but without our technique, the amount of dimensionality reduction to use is difficult to predict ahead of time and can depend on the hand and task. The models we produce can be used to plan and carry out successful, robust manipulation actions and to compare competing robot hand designs.

## 1. Introduction

In industrial applications, robotic systems have been successful for decades for certain assembly line tasks such as spot welding (Wang and Guu, 1989). The motivation for automating these tasks comes from the high cost of skilled human labor and the serious risk of injury present in many manufacturing processes. Today, the goals of modern robotics extend beyond the rigid structure of the factory environment, seeking to safely and robustly perform in the relative chaos that is everyday life.

An emerging class of robots designed to address this problem are soft robots made from *intrinsically* soft materials (Deimel and Brock, 2013; Tang et al., 2019; Zhang et al., 2019; Zhou et al., 2019; Li et al., 2020). Apart from being safe, the softness and compliance realized by these robots can be exploited to reduce the complexity of environmental interactions. For example, the compliance of soft robots allows them to adapt to geometric variations without the need for complex low-level control, a feature shared by robot hands which contain rigid skeletons but may have compliant joints or actuation (Dollar and Howe, 2009; Odhner and Dollar, 2011; Xu and Todorov, 2016; Della Santina et al., 2018; Homberg et al., 2019). Such an exploitation of compliance can be observed in many biological organisms and is therefore a promising characteristic (Majidi, 2014). Soft robots have shown great ability to conform to object surfaces, produce stable grasps, and handle fragile objects gently (Brown et al., 2010; Galloway et al., 2016; Stuart et al., 2017). However, achieving human-level dexterity, including dexterous in-hand manipulation, remains a challenge. Enabling such capabilities requires considerable innovation in hardware, modeling, and control (Marchese and Rus, 2016; Zhou et al., 2018; Abondance et al., 2020).

**Figure 1:**
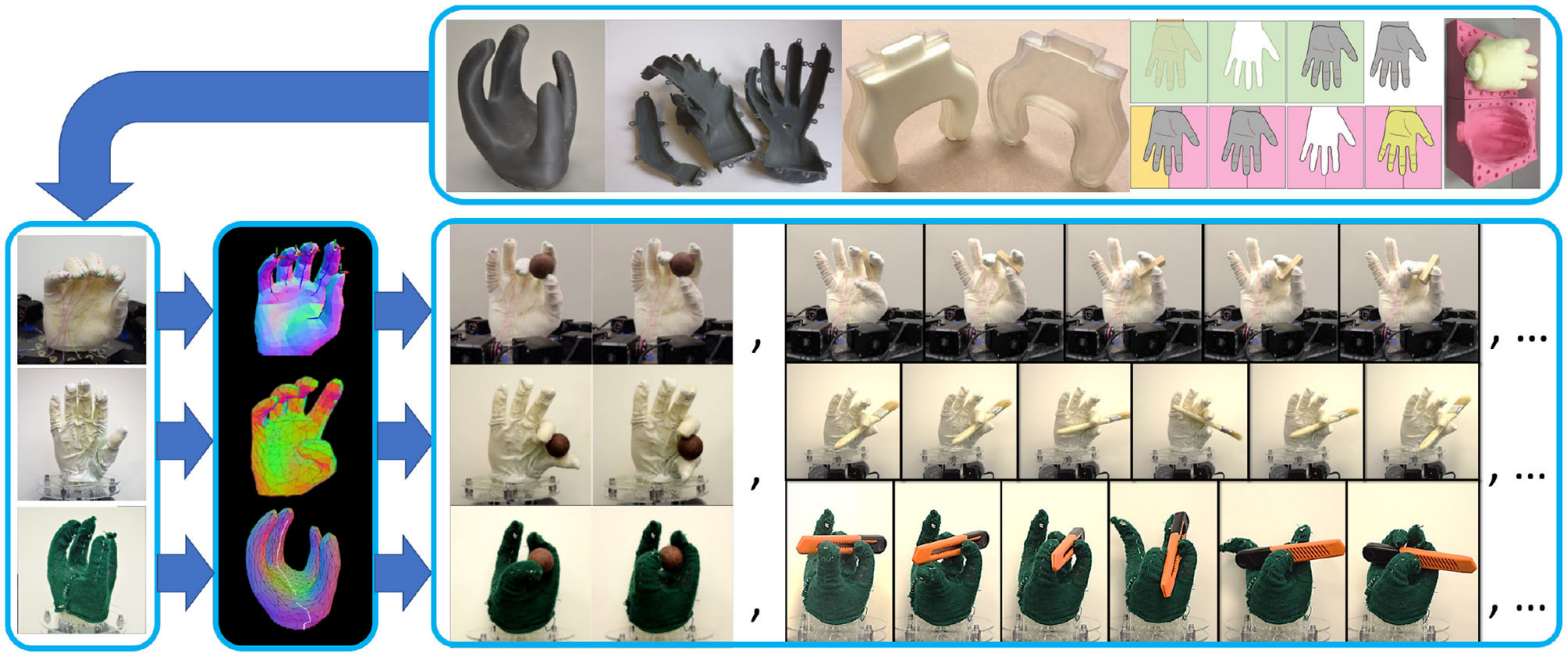
From the top in the direction of the arrows we show: (1) Highly diverse, highly complex hand morphologies can be rapidly and in-expensively fabricated using simple 3D-printing and casting methods. (2) The hands are driven using embedded tendons integrated with the foam via textile skins. (3) Customized, interactive soft-body simulators allow for exploration of designs, actuator configurations, and planning strategies. (4) A variety of complex and dexterous manipulations can be performed across a wide range of hand designs, demonstrating the capabilities, and tremendous potential of soft foam manipulators.

One very promising approach is to design low-cost, easily manufacturable hand designs which can be customized for specific tasks. For example, King et al. (2018) present a class of novel fully-compliant, tendon-driven soft hands made from off-the-shelf components (Figure 1). The primary structure of this type of robot consists of a flexible foam core. The softness and flexibility of the foam hand have been shown to be of great advantage for stable and secure grasping, and robust in-hand manipulation (King et al., 2018). However, these hands do have trade-offs. The advantages gained by softness come at the cost of necessarily more complex control and planning strategies. This is due to the uncertainties created by complex contact mechanics, deformable materials, and the lack of a compact Forward and Inverse Kinematics representations (i.e., solve FEA instead of using rigid body transformations with joint angles). Another trade-off is that soft robots have a lower upper-bound on the velocities and forces they can operate at due to their low stiffness, thus may not be suitable for high speed or high force industrial processes. However there are very complex manipulations that do not require high speeds or high loads, such as tasks done day-to-day by human hands. At these kinds of speeds we have not observed performance issues with the kinds of soft hands shown in this paper.

The goal of this research is to provide an intermediate representation of soft hand capabilities to facilitate control and planning, as well as to discover the inherent competence or dexterity of a given hand design. Specifically, we present an approach to identify and model continuous families of manipulation actions for these robot hands. The models described in this paper can be used directly for planning and control, as successful actions can be sampled from the model or combined in a planner. A planner that has access to a large family of strategies to achieve a manipulation goal can be robust to different environments: if it cannot achieve its goal with its primary strategy, rather than being stuck with failure, it can explore secondary planning strategies and beyond. We note that some manipulation actions are multimodal; in developing our models, we identify discrete different ways in which target manipulations can be accomplished. Identifying and separating multiple modes of performing manipulations provides a valuable tool for both planning and local control. A planner, for example, can attempt to formulate a plan using each potential mode and select the option with greatest utility for the current scenario.

These models presented in this paper also provide a representation of a robot hand's capabilities. This representation is in terms of the space of actions that produce successful manipulations. A large action space indicates substantial flexibility in performing a manipulation action—that manipulation can be performed by the robot hand in many varied ways. If a hand has a large, continuous successful action space for a manipulation, it may be fair to classify that manipulation task as “easy” for that hand design. As such, these models can be used to compare hands and refine designs. Hand design parameters can be tuned and optimized, or discrete design elements can be selected by measuring their effect on the size of the action spaces for manipulations that are of particular interest for the robot hand under development.

Finally, we present a pure intellectual curiosity behind this work—given a new hand design, we are driven to see just what that design may be able to accomplish. Our approach provides an empirical means to begin to achieve that goal and quantify the results.

## 2. Related Work

The continuously deformable nature of soft robots makes controlling them a challenging problem. Their ability to accomplish motions such as buckling, extension, or bending, results in soft robots having virtually infinite degrees of freedom. Additionally, George Thuruthel et al. (2018) mention nonlinear material effects such as compliance, visco-elastic material behaviors, and hysteresis, as well as the wide range of design and actuation techniques that account for the non-trivial nature of this problem. Previous works have particularly studied the problem of inverse kinematics (IK) which is concerned with finding a mapping between actuator configuration and desired hand configuration (i.e., pose) (Rolf and Steil, 2013; George Thuruthel et al., 2016; Jiang et al., 2017; Schlagenhauf et al., 2018; Bauer et al., 2020). Existing control approaches can be classified into three main categories: model-based or model-free controllers, as well as combinations of both. The model we refer to here follows the definition of model by Sutton and Barto (2018), that is ‘a model of environment, something that mimics the behavior of the environment, or more generally, that allows inferences to be made about how the environment will behave'.

Model-based controllers rely on the establishment of a kinematic mapping from which the actuation can be directly inferred for the desired configuration. A popular approach is to use lumped parameter models and pseudo rigid-body models (Saunders et al., 2010), model caterpillar-like soft robots as a series of extensible linkages. For tentacle-shaped soft robots, Marchese et al. (2014), Marchese and Rus (2016), and Chen et al. (2013) use piecewise constant curvature models to model the robot. For soft robots with arbitrary shapes, Duriez (2013) presents a real-time solution using a finite element method (FEM).

Model-free approaches offer a wide variety of data driven techniques to control soft robots. Neural networks have successfully been used to learn inverse kinematics on a cable driven soft tentacle manipulator with 2 degrees of freedom (Giorelli et al., 2015). Rolf and Steil (2013) have proposed an exploration algorithm for creating task space samples for IK learning. Model free approaches have been successfully used to learn grasping and manipulation tasks for soft hands from demonstration (e.g., Gupta et al., 2016; Della Santina et al., 2019).

Our approach is model-free in the sense that while we sample data from simulation, we do not reason explicitly about the system configuration. Instead we observe the effect of an action toward achieving an intended manipulation (e.g., reorientation of an object within the robot hand).

The challenge of working with data of this sort is three fold. First, the sample space is usually large in dimension, so we need efficient sampling methods. Second, we must determine which degrees-of-freedom, or dimensions, are significant. And third, we require appropriate models for representing planning strategies.

We have identified solutions to these problems by using well known methods like stratified sampling (Mitchell, 1996; Dellaert et al., 1999), principal component analysis (Jolliffe and Cadima, 2016), and Gaussian Mixture Models (GMM) (Reynolds, 2009), to tackle the problems of sampling, significant dimension determination, and modeling approach, respectively.

Prior art has utilized similar building blocks. For example, Khansari-Zadeh and Billard (2011) use Gaussian Mixtures to ensure local asymptotic stability of dynamical systems, Nguyen-Tuong et al. (2008) use Gaussian Process Regression to achieve real-time online model learning, Calandra et al. (2016) use Manifold Gaussian Processes on non-smooth complex functions such as contact mechanics, Bidan Huang et al. (2013) use GMMs to generate grasps given a starting object-hand configuration, Lawrence (2005) use Dual probabilistic PCA and Gaussian Processes for dimensionality reduction, and Engel et al. (2005) use Gaussian Processes with Temporal Differences to learn a controller for a soft octopus tentacle. What distinguishes our approach is that we utilize GMMs to form *global models* of *successful action spaces*. Action spaces can be modeled in many different ways, and we give results from two concrete examples in this manuscript. However, for any model, we aspire to capture the entire valid space of possible manipulations as one or more continuous action regions.

## 3. Method

We propose a sampling based method to identify and model families of manipulations for soft robot hands. Given a manipulation goal for a soft hand, our approach aims to discover and model large continuous action spaces that allow the manipulation goal to be successfully accomplished.

We elaborate on the design of our method in the sections below. In section 3.1, we explain how we represent the physical environment and the soft hand's interaction with that environment, including defining manipulation goals and action spaces. In section 3.2, we unfold our three-step method for generating models of continuous successful action spaces to achieve manipulation goals. Experiments used to test the validity of our method are discussed in section 4.

### 3.1. Problem Definition

In this section, we formally define the soft robot hand's actions, states, and manipulation goals. Table 1 contains a listing of variables and their descriptions. Specific instantiations of these variables are given for two robots in sections 4.1 and 4.2.

**Table 1:** Variable Table.

**Variable**	**Description**
***θ***	Single parameter sample, defining an open loop action for the foam hand
action space	Continuous region of parameters ***θ*** that may achieve some goal (e.g., result in performing a manipulation task successfully)
Θ	Set of parameter samples ***θ***
Ω	Parameter space from which ***θ*** can be sampled for a foam hand
*d*	Dimension of Ω
*ϕ*(***θ***, *t*)	Controller function, which produces an action from parameter ***θ*** and time *t*
***a***_*t*_	Action performed at time *t*, defined as an open loop control actuation command
*d*_*a*_	Dimension of an action; degrees-of-freedom in the action space for a single time *t*
***s***_*t*_	State of the observed physical system (including manipulated object) at time *t*
***s***_*goal*_	Goal state of the observed physical system.
*d*_*s*_	Dimension of the state vector; observed degrees-of-freedom in the physical system
*score*	Weighted difference between observed state and goal state in a manipulation
*b*	Coefficient vector weighting state differences for computing a manipulation score
*c*_*minScore*_	Minimum score required to consider one parameter ***θ*** as successful.
*n*_*initial*_	Target number of successful samples to collect in the initial sampling process
*n*_*sample*_	Number of samples to test in one iteration of the initial sampling process
*s*	Scale factor *s* > 1 controls exploration beyond observed successes in initial sampling
*m*	Number of PCA dimensions for a proposed GMM, *m* ≤ *d*
*k*	Number of Gaussians for a proposed GMM
*p*(***θ***) or *GMM*_*m, k*_	Gaussian Mixture Model built over Θ with hyperparameters m, k
*p*′(***θ***)	GMM whose Gaussians have normalized inverse weights of *p*(***θ***)
ϵ_*w*_*std*_	Threshold to achieve sufficiently uniform GMM weights
ϵ_*w*_*diff*_	Threshold to indicate progress is not being made on equalizing GMM weights
ϵ_*succ*_	Threshold to indicate a match for desired GMM success rate
***w***_*old*_	Old weight of Gaussians constructed in the model building process
***w***_*new*_	New weight of Gaussians constructed in the model building process
*c*_*succ*_*rate*_	Target (required) success rate for each proposed GMM

We start with the definition of actions of a soft robot hand. We assume that the soft robot hand is driven by its controller. The controller takes a parameter vector ***θ*** as input. ***θ*** corresponds to attributes that could be used to generate a control signal, things like positions, joint angles, forces, torques, as well as their combinations. This parameter vector is drawn from a parameter space Ω which is established ahead of time by the user. The specific definition of the parameter space depends both on the robot hand design and the choice of control. For example, one possible set of parameters is a list of actuator commands and associated timestamps, which could be used to actuate the hand with an open-loop PD controller.

Given a parameter vector ***θ***, the controller generates a sequence of open-loop actuation commands called actions, $a_{t=1\ldots T}\in {\mathbb{R}}^{d_{a}}$, where *d*_*a*_ represents the number of controlled degrees-of freedom. The controller actuates the foam hand by executing the action generated by ***a***_*t*_ = *ϕ*(***θ***, *t*) for all *T* time steps.

As the soft foam hand's controller executes its actuation commands, its surrounding physical environment would be affected by its behavior. For example, the action may result in manipulation of a grasped object. To represent this change in the system, we define the state of the physical system containing the soft hand for any time step *t* as a vector, $s_{t=1\ldots T}\in {\mathbb{R}}^{d_{s}}$, where *d*_*s*_ represents the number of observed degrees-of-freedom. The physical system always starts with a rest state ***s***_0_ and ends with a final state ***s***_*T*_. The state vector contains numbers which correspond to measurable attributes of the scene at a given time step such as positions, velocities, forces, or even raw sensor values.

The objective of the controller is for the final state to be ***s***_*goal*_. At each time step *t*, the foam hand controller can interact with the system by executing ***a***_*t*_, which transforms the system according to the state update function, ***s***_*t*+1_ = simulator(***s***_*t*_, ***a***_*t*_). After the controller finishes executing the actions, we compare the final state of the system ***s***_*T*_ against the goal ***s***_*goal*_ to evaluate ***θ***'s effectiveness in manipulating the soft hand. Specifically, we generate a score, *score* = ***b*** · ||***s***_*T*_ − ***s***_*goal*_||, taking the inner-product of a linear coefficient vector, ***b***, and the normed error vector, ||***s***_*T*_ − ***s***_*goal*_||.

To classify the parameter vector ***θ*** used by the controller to generate actions, we compare *score* against a minimum score threshold, *c*_*minScore*_, where *successful* or *failure case* occurs if *score* is above or below the minimum threshold, respectively. In practice, this is evaluated using the conditional indicator function:

$$𝟙(score>c_{minScore})=\{\begin{array}{ll} 1 & \text{if }\text{True}\\ 0 & \text{if }\text{False} \end{array}$$

Therefore, in the later section of this paper, when we mention *successful* parameters, we refers to those that satisfies the indicator function 𝟙(*score* > *c*_*minScore*_). On the contrary, *failed* parameters have scores below the threshold *c*_*minScore*_.

The goal of the research described in this paper is to identify large continuous regions of parameters ***θ*** that result in achieving the user specified goal ***s***_*goal*_. In other words, we wish to find large continuous regions of ***θ*** that are classified as successful. We informally call these regions action spaces as they represent continuous families of actions that the soft robot hand can take in order to achieve the desired result.

**Algorithm 1:** GMM Model Construction Pipeline.

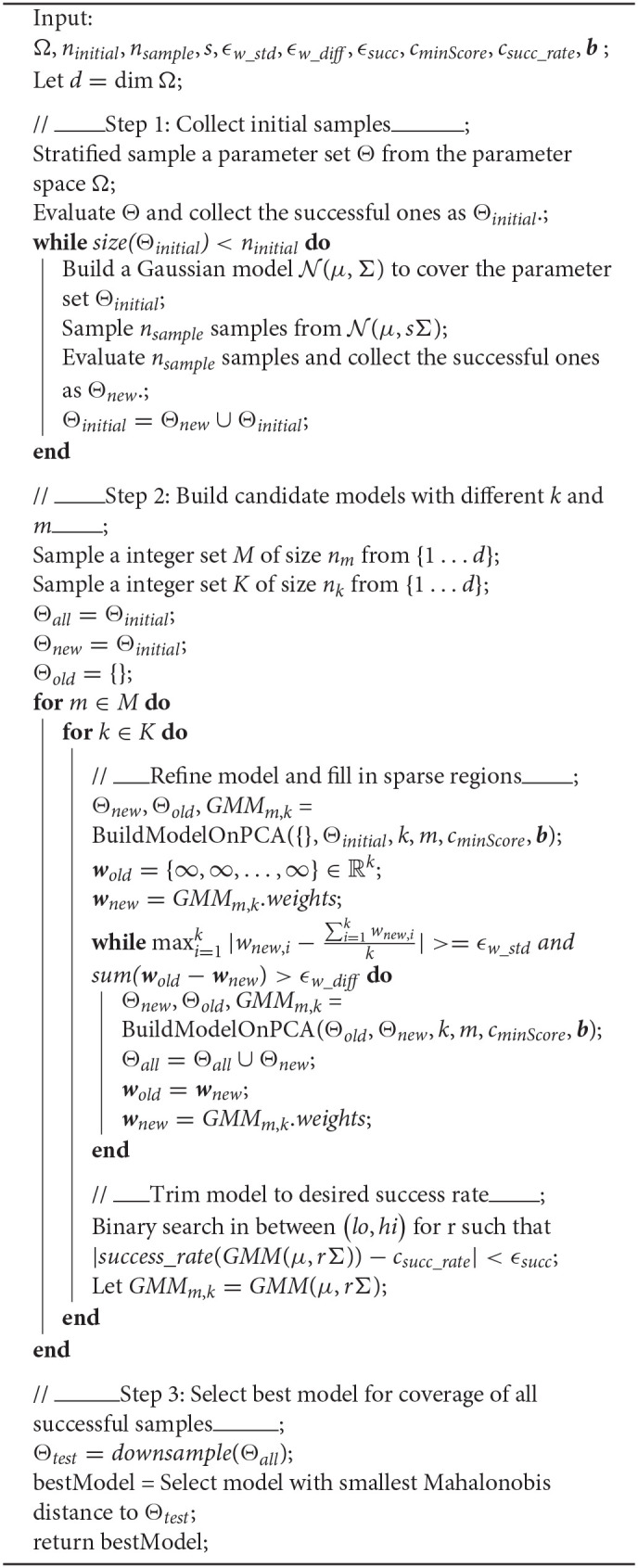

**Algorithm 2:** BuildModelOnPCA(Θ_*old*_, Θ_*new*_, *k, m, c*_*minScore*_, *b*).

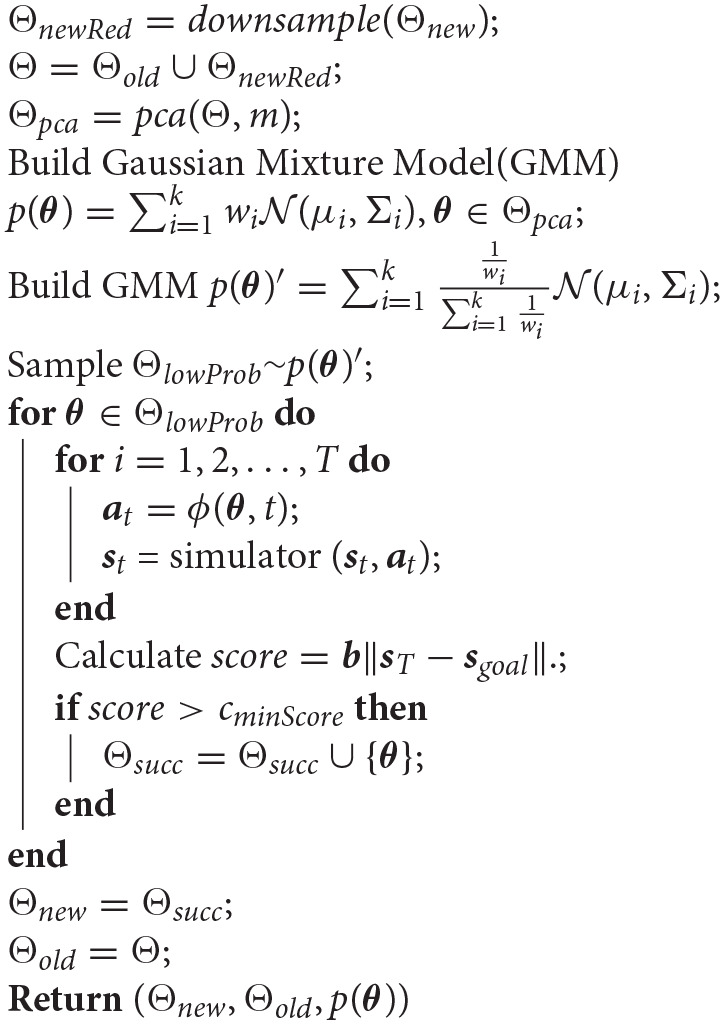

### 3.2. Approach Overview

Our method is designed to identify and model continuous action spaces that allow a soft robotic hand to achieve a manipulation goal successfully. The resulting models are intended for use in manipulation control, manipulation planning, and robot hand design/evaluation.

At high level, our method can be considered as black box that takes in all the physical system information, for example, goal state ***s***_***goal***_ and initial state ***s***_***initial***_, and soft hand action information, such as parameter space Ω and controller *ϕ*, then builds a model to cover the *successful* parameter distribution in Ω, eventually outputting a model to generate parameters for soft hand controllers to achieve their objective of changing their surrounding environment's state to ***s***_***goal***_.

**Figure 2:**
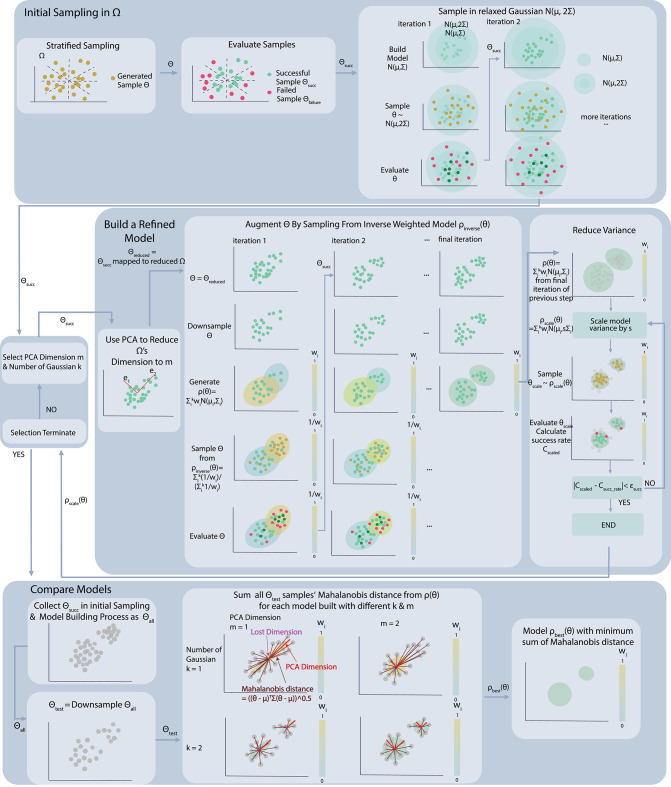
Algorithm flow chart.

Specifically, we employ a sampling based approach consisted of three steps, which are shown in Figure 2: (1) collect a large number of successful samples of performing the desired manipulation, (2) construct a collection of alternative models, refining each model to “fill-in” sparsely sampled regions and achieve a target success rate, and (3) compare all models for best coverage of an unbiased test dataset formed by selectively downsampling all successful samples ever seen. The approach terminates by selecting and returning the best model. Pseudocode for the full pipeline can be found in Algorithm 1. Each of the three steps will be discussed in detail in sections 3.3–3.5, respectively.

### 3.3. Collect Data in the Full Dimensional Parameter Space

The first step of the algorithm is to collect a large set of successful manipulations. To achieve this goal, we use a two-step process, first collecting data by sampling within the full action space using stratified sampling, and then performing a more focused exploration in the area “near” successful samples that have already been identified.

An initial exploration of the action space is performed using stratified sampling (Mitchell, 1996). The stratification is done by dividing the parameter space Ω ∈ ℝ^*n*^ into small sub-regions by subdividing each of the *n* dimensions. Samples are drawn randomly from within each subregion to ensure some coverage of the entire space. This approach helps induce improved sampling uniformity even with a relatively small sample size.

Once an initial collection of successful samples have been drawn, the search is focused to an area where successful samples are more likely to occur, i.e., we switch to a process of sampling with constraint. The constrained space is constructed by building a Gaussian Model from the existing collection of successful samples, $M_{initial}=N\left(\mu ,\Sigma \right)\sim \Theta$, where N(μ,Σ) is a standard Gaussian where parameters Θ represent successful samples.

To facilitate some exploration within the constrained sampling process, We create a new model N(μ,sΣ) whose covariance matrix *s*Σ is equivalent to *M*_*initial*_'s covariance matrix scaled by a scalar *s* > 1. By sampling from this relaxed Gaussian model N(μ,sΣ), we increase the probability of collecting successful parameters compared to the original parameter space Ω, without limiting the new sampling domain to only the successful cases we have encountered thus far. The examples in this paper use *s* = 2. We iteratively construct relaxed Gaussian models on the sample set Θ collected *so far* and sample from them until we pass *n*_*initial*_, the minimum number of samples required during the initial sampling process.

### 3.4. GMM Construction

After collecting a suitable number of samples, we move to the next step of the algorithm, the central box in Figure 2. The goal of this step is to generate a candidate set of models of the continuous successful action space. The models are Gaussian Mixture Models, and they vary in two parameters: the number of Gaussians selected (*k*) and the dimension of the space in which they reside (*m*).

The motivation for testing different numbers of Gaussians (*k*) is fairly obvious. We wish a representation of our action space that is both compact and comprehensive. As such, we would like to identify the smallest number of Gaussians that covers the continuous successful regions of the action space well (i.e., without missing large successful regions). It is difficult or impossible to predict this number ahead of time, and so we test different values.

The motivation for testing spaces of different dimension (*m*) is perhaps less obvious. It has been shown repeatedly that many actions that appear complex are in fact highly coordinated (e.g., Safonova et al., 2004; Ranganath et al., 2019). It seems likely that some reduced dimensional space would be adequate for representing the entire successful action family. However, how many dimensions may be needed is again difficult to predict. Here again, we test different values.

The output of this step is a collection of models having different *k* and *m* values and an extended set of successful samples, all of which are used to determine a final best model in the third and final step. The next section details the model building process for an individual *k* and *m* value, assuming that these values have already been selected.

#### 3.4.1. Dimension Reduction With PCA

We now walk through the process of building and refining a single model (the central block in Figure 2). The first step is to identify a suitable reduced dimensional space of size *m*. For this, we use PCA (Jolliffe and Cadima, 2016), keeping the first *m* eigenvectors of the projection *P* as the basis of the space *R* with reduced dimensions. There are many ways we could take a lower-dimensional projection of our space. PCA is a favorable choice because, by definition, it is an orthogonal linear transformation which maximizes the variance of the projected data. Essentially, all of the *significant* structure is maintained while reducing its span to a volume more tractable for sampling and computation.

$$\begin{array}{l} \;R=Q\Omega^{*}\\ \;C_{\Omega }=\frac{1}{n}\Omega^{*T}\Omega^{*}\\ \;=Q\Lambda Q^{T} \end{array}$$

Note that to extract the principal components with regard to the covariance of Ω, we first shift Ω to be a “0-centered” space Ω^*^, taking the origin to be its mean. Then, we make an eigen-decomposition on the covariance of the centered parameter space Ω^*^. The principle components of Ω^*^ are what we call the eigenvectors of the covariance matrix*C*_Ω_ and it is used to transform the basis of the original space Ω to the reduced space *R* (Jolliffe and Cadima, 2016).

#### 3.4.2. Bias Reduction: Downsampling and Inverse Model

Even with PCA, if we build a model directly on all of the data accumulated in Θ, it would create bias. This bias arises due to the accumulated data containing disproportionate numbers of successes drawn from the initial stratified sampling process. This mean-offset would result in an advantage for the “first-come” data that decreases the likelihood of discovering disjoint strategies. To eliminate this, we downsample Θ. Specifically, we greedily select a subset $\Theta_{sub}=\text{arg}{\text{max}}_{\Theta_{sub}\subseteq \Theta ,|\Theta_{sub}|=n_{sub}}std(\Theta_{sub})$, that has the largest subset among Θ's subsets of same size *n*_*sub*_. In this way, we do not favor frequently sampled data, instead encouraging a narrowing of the success frequency-gap.

To further reduce bias in collected samples and allow sparsely populated regions to be “filled-in,” we draw new samples from an “inverse GMM model” built on the downsampled data. Specifically, we construct the original GMM model with the usual expectation maximization algorithm similar to the k-clustering process. Then we create a “inverse model” whose weight $w_{i}^{\prime }$ for its ith Gaussian is $\frac{1}{w_{i}}$, which means that the Gaussian of highest weight would be the one with lowest weight in the “inverse model.” This step is based on the assumption that all of the successful samples/actions should be equally good in terms of performing the target manipulation tasks. Thus, each cluster in the space Ω should be considered as equally important. Correspondingly, Gaussian components of our Gaussian Mixture Model representation, corresponding to clusters in the parameter space, should have evenly distributed weight *w*_*i*_. We used the method of reducing the samples for high weight Gaussians and increasing the samples for low weight Gaussians to balance out the unevenness in the Gaussian weights by encouraging exploration within Gaussian regions that have low probability in the original model.

**Figure 3:**
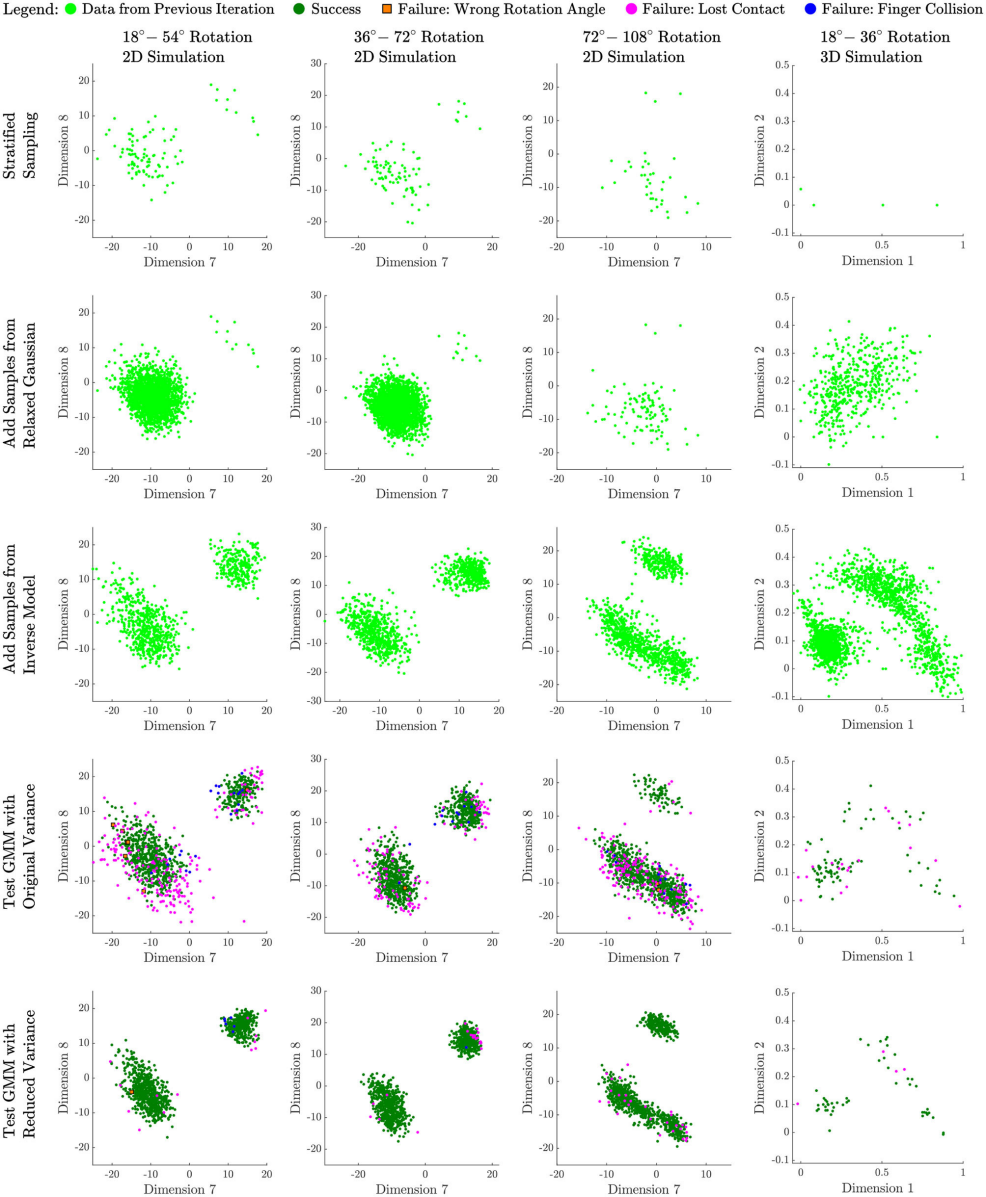
Successful experiments from left to right: Data evolution for our best models using a 2D simulation with angle rotation ranges of 18–54, 36–72, and 72–108 degrees, and a 3D simulation with angle rotation range of 18–36 degrees.

We continue this iterative process of reducing bias, by repeatedly reducing dimension of downsampled data with PCA, building a model on the data with reduced dimension, creating an inverse model and sampling new data from the inverse model, downsampling the results and adding them to the downsampled set of data accumulated so far. This process is illustrated in Figure 2, which shows graphically how an undersampled region covered by a low-probability Gaussian can be populated through the process and brought to nearly equal probability to the Gaussian containing the larger portion of “first-come” data. A similar phenomenon illustrated with actual data is shown in rows 2 and 3 of Figure 3, where a sparsely populated secondary region becomes evenly populated after the inverse model sampling process.

The iterative process terminates when the difference between Gaussian Components' weight of is small enough that $\overset{k}{\underset{i=1}{\max}}|w_{i}-\frac{\overset{k}{\underset{i=1}{\sum }}\left(w_{i}\right)}{k}|<\epsilon_{w\text{\_}std}$. Occasionally, if the number of Gaussians is not a good match for the geometry of the successful parameter space, this process may be slow to terminate. In that case, we watch for small changes in weights and finally terminate at a maximum total number of iterations (see Algorithm 1).

#### 3.4.3. Reduce Variance to Elevate Success Rate

So far, models have been constructed based on successful samples only. However, the GMM that results will also contain failures. We observe that these failures are typically on the boundaries of the modeled regions. To reduce failures and bring success rate to a desired level, we propose a simple strategy of variance reduction.

Specifically, to increase the final success rate of the model, we linearly scale the covariance matrix Σ for each component Gaussian of the GMM. We use binary search to search for a scale rate *r* such that model *GMM*_*m, k*_ = *GMM*(μ, *r*Σ) would have success rate of approximately *c*_*succ*_*rate*_ when the samples drawn from it for planning are tested either on the physical soft hand or in simulation. Models which cannot achieve the desired success rate after a variance reduction of 0.2 are discarded. Over many experiments, only one model had to be discarded due to this criterion, indicating that the assumption of failures occurring on the boundaries of the Gaussian regions is effective in practice.

### 3.5. Compare and Select Models

In section 3.4, we explained how an individual model with a fixed *m* and *k* value is being created. In this section, we want to elaborate on how *m* and *k* would be tuned to create the model that best fit the space.

Ideally, we would perform a global search over all plausible parameters *m* and *k*. There are multiple ways of doing such a search of optimal parameters. However, empirically, what we found to work well was to do a line search over PCA dimension *m*, followed by a line search over *k*. After extensive informal experimentation, we found that this process was efficient and tended to produce a nearly optimum, if not the optimum model.

#### 3.5.1. Squared Distance Between Θ_*test*_ and the GMM

The final step of the process (bottom section of Figure 2) is to compare models and select the bst one. We compare models based on their coverage of all of the successful test data seen so far. To evaluate the parameter space coverage of different models, we create a test data set Θ_*test*_ by downsampling all successful data. The downsampling method is the same one used in section 3.4.2. The model that fits this data set the best should be the model that covers the greatest amount of variance we have explored so far in the parameter space Ω.

However, it is challenging to directly compare our models' coverage of the test dataset, since they all have different PCA dimension *m*. To account for the difference in models' dimension, We need a variance comparison practice that encourages models to preserve components of the test data that are within their dimension-reduced space, while penalizing low-dimensional models for reducing too much variance simply as a result of the projection step involved in dimensionality reduction.

Thus, correspondingly, we measure the model's variance coverage by combining “in-model distance” and “out-of-model” distance from each data in the test set. To check the fitness of a model, we would project each test data point in the data set into the model's dimension reduced space of size *m* and then calculate its Mahalanobis Distance from each Gaussian of the model. Its minimum Mahalanobis Distance from the Gaussian would be considered as its “in-model distance” from the model. On the other hand, to measure the lost variance of a dimension-reduced model, we construct a Gaussian that tightly covers the test data in the full parameter space and calculate the test data's Mahalanobis Distance from this Gaussian in the “lost” dimensions only. This distance forms the “out-of-model” distance. The equations describing the distance metric we use are as follows, where ⊙ is the element-wise matrix product.

$$\begin{array}{l} Q_{red}=[q_{1}|q_{2}|\ldots |q_{m}]\\ Q_{lost}=[q_{m+1}|q_{m+2}|\ldots |q_{n}]\\ \text{ where }q_{i}\text{is the }i\text{th column vector of }Q\\ \text{ and m is the number of PCA dimensions that}\\ \text{ we reserve in the model}\\ \;\theta^{*}\text{ = }\theta -\mu_{\Omega^{*}}\\ \text{ where }\mu_{\Omega^{*}}\text{ is the shift we used to translate space }\\ \;\Omega^{*}\text{ to space Ω}\\ f(\theta^{*})=Q_{red}^{T}\theta^{*}\\ g(\theta^{*})=Q_{lost}^{T}\theta^{*}\\ \bar{\mu }(\Theta_{test})=\frac{\sum_{\theta^{*}\in \Theta_{test}}g(\theta^{*})}{\left|\Theta_{test}\right|} \end{array}$$

$$\begin{array}{l} \bar{\sigma }(\Theta_{test})=\sqrt{\frac{\sum_{\theta^{*}\in \Theta_{test}}(g(\theta^{*})-\bar{\mu }(\Theta_{test}))⊙(g(\theta^{*})-\bar{\mu }(\Theta_{test}))}{\left|\Theta_{test}\right|}}\\ {\text{Σ}}_{lost}=\text{diag}(\bar{\sigma }(\Theta_{test}))\\ SS\left(\sum_{i=1}^{k}w_{i}N(\mu_{i},{\text{Σ}}_{i})\right)=\\ \;\sum_{\theta^{*}\in \Theta_{test}}(\overset{k}{\underset{i=1}{\min}}\left({\left(f(\theta^{*})-\mu_{i}\right)}^{T}{\text{Σ}}_{i}^{-1}(f(\theta^{*})-\mu_{i})\right)\\ \;+\;{\left(g(\theta^{*})-\bar{\mu }(\Theta_{test})\right)}^{T}{\text{Σ}}_{lost}^{-1}(g(\theta^{*})-\bar{\mu }(\Theta_{test}))) \end{array}$$

The sum of squares of each test data's “in-model distance” and “out-of-model distance” forms the total squared distance between a GMM and the test data set. The larger is this total distance, the weaker the model is in covering the parameter space Ω. Therefore, the model with the minimum total distance has the optimal *k* and *m* value that results in the best coverage of variance of the original parameter space.

## 4. Results

### 4.1. 2D Simulation of a Soft Hand

**Figure 4:**
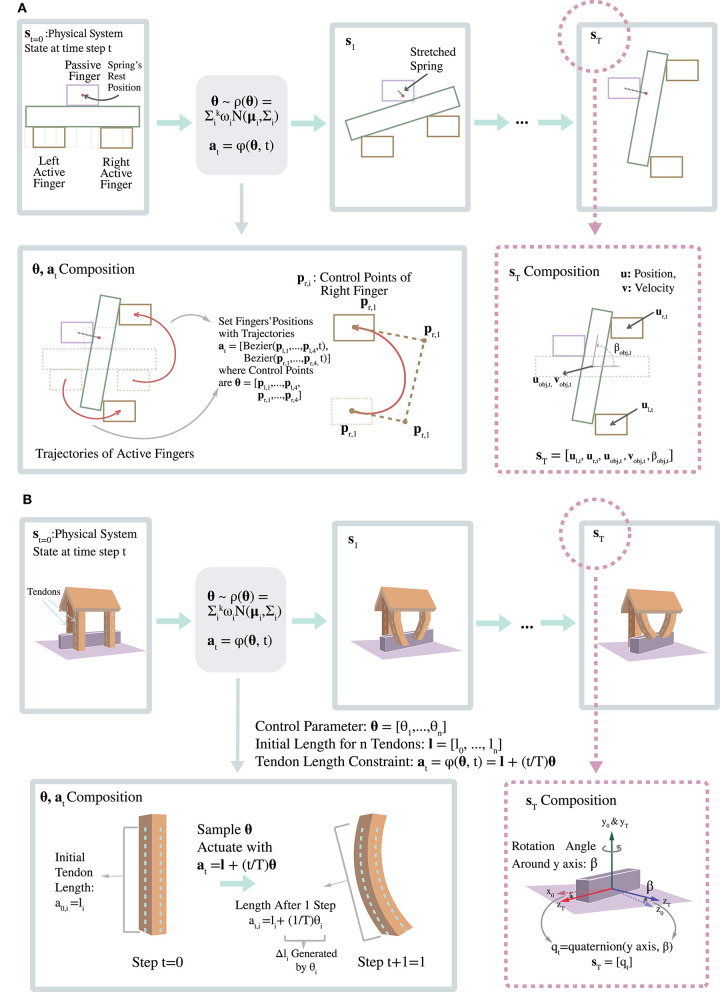
Illustration of 2D and 3D Rotation Tasks (top and bottom, respectively).

To test the effectiveness of our method, we design a 2D simulation of a three-finger soft hand. The manipulation goal for the soft hand is 2D rotation of a rigid-body object. Thereby, to fit the manipulation goal, we set up an environment that emulates the general situation that a human hand tries to rotate a thin and elongated object, for example, a pen, in mid air. The setup for the 2D hand is illustrated in Figure 4 (top).

The state of the system at time step *t* is represented by the vector ***s***_*t*_ = [*x*_*l, t*_, *y*_*l, t*_, *x*_*r, t*_, *y*_*r, t*_, *x*_*obj, t*_, *y*_*obj, t*_, *v*_*ob*_*j*__*x*_, *t*_, *v*_*ob*_*j*__*y*_, *t*_, *β*_*obj, t*_], which records the position of left finger tip ***u***_*l, t*_ = [*x*_*l, t*_, *y*_*l, t*_], the position of the right finger tip ***u***_*r, t*_ = [*x*_*r, t*_, *y*_*r, t*_], the position and the velocity of the center of mass of the rotated object, ***u***_*obj, t*_ = [*x*_*obj, t*_, *y*_*obj, t*_] and ***v***_*obj, t*_ = [*v*_*ob*_*j*__*x*_, *t*_, *v*_*ob*_*j*__*y*_, *t*_], and the rotated angle of the rigid object *β*_*obj, t*_.

The action command at time step t is defined as the concatenation of the two active fingers' positions ***a***_*t*_ = [*u*_*l, t*_, *u*_*r, t*_]. The action sequence for two fingers is generated by two cubic Bezier splines, each of which takes four control points as its input. Thus, the action is parameterized by $\theta =\left[p_{l,1},p_{l,2},p_{l,3},p_{l,4},p_{r,1},p_{r,2},p_{r,3},p_{r,4}\right]\in {\mathbb{R}}^{16}$, which is a vector containing all eight control points ***p***_*f* = {*l, r*}, *i* = {1, …, 4}_ to create two cubic Bezier curves for two active fingers. The corresponding action generation function is ***a***_*t*_ = [*Bezier*([***p***_*l*, 1_, ***p***_*l*, 2_, ***p***_*l*, 3_, ***p***_*l*, 4_], *t*), *Bezier*([***p***_*r*, 1_, ***p***_*r*, 2_, ***p***_*r*, 3_, ***p***_*r*, 4_], *t*))].

$$\begin{array}{l} Bezier\left(\left[p_{1},p_{2},p_{3},p_{4}\right],t\right)={\left(1-t\right)}^{3}p_{1}+3{\left(1-t\right)}^{2}tp_{2}\\ \;+3\left(1-t\right)t^{2}p_{3}+t^{3}p_{4},\;0\leq t\leq 1 \end{array}$$

Eventually, in the 2D simulation, we want to find families of successful rotation trajectories generated from continuous families of parameter ***θ***. In this experiment, we explore the parameter space using the sampling based approach outlined in section 3, Algorithm 1, and Figure 2. Based on this setup of 2D simulation, if we can observe a variety of successful rotation actions being generated via our method, than we achieve our goal of creating diverse manipulation strategies for soft hands.

To examine our methods' ability to capture families of strategies for a soft foam hand, we set up three experiments to enable the simulated 2D hand to rotate a slender cuboid shaped object over different ranges of angles around an axis parallel to our line of sight. Those ranges are A:(18–54), B:(36–72), and C:(72–108), with all angles measured in degrees.

**Figure 5:**
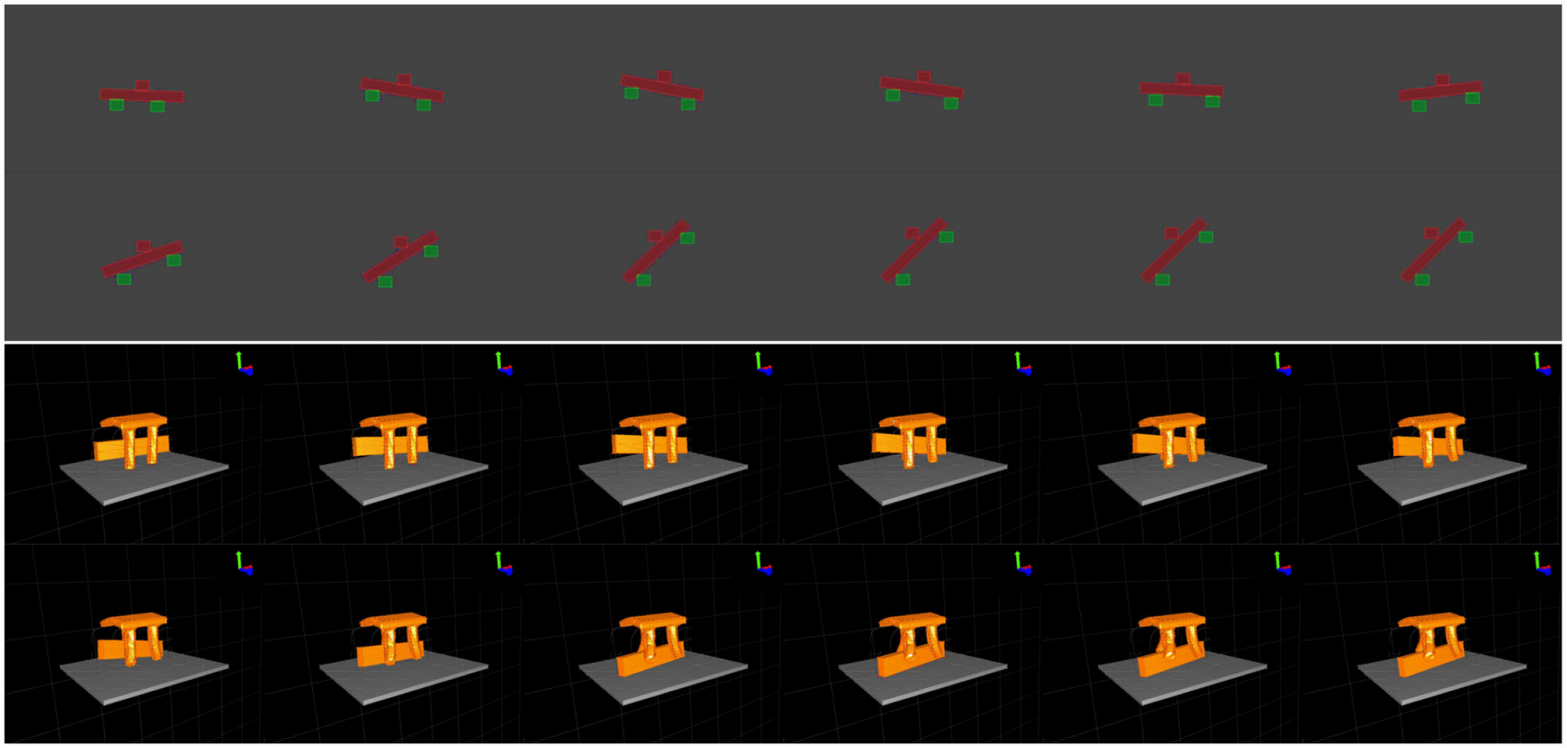
From left to right and top to bottom, frames captured from 2D and 3D simulated manipulations.

Criteria for success specifically were (1) all fingers must be in contact with the rod at the end of the action sequence, (2) the final rotation angle falls in the designated range, and (3) there are no collisions between fingers in the action sequence. A filmstrip illustrating a successful manipulation is shown in Figure 5.

During initial sampling, which included both stratified sampling and collecting samples from the relaxed Gaussian, 1,990, 2,436, and 102 successful samples were collected for tests A, B, and C, respectively. The number of successful samples for test C is much lower in the initial steps due to the relative difficulty of the task. The final number of successful samples that were collected as a result of all of the model building processes was 31,250, 27,172, and 28,078 for tests A, B, and C, respectively. Running times were approximately 5.5 h for collecting initial samples and 1.6 h for building, refining, and reducing variance for a single choice of model with a single choice of *m* and *k* on a 1.4 GHz Quad-Core Intel Core i5.

Results for three different 2D simulation tests are shown in Table 2. Each section of the table gives results for models having different numbers of PCA dimensions *m* and Gaussians *k*. The table shows the amount by which variance had to be scaled in order to reach the required success rate. It also shows the Mahalonobis distance of the resulting model for fitting the test dataset.

**Table 2:** Simulation results identifying the best models.

**PCA Dim. m**	**Num. Gaussians k**	**Variance Reduction Scale r**	**Squared_Distance**
**2D simulation A: 18–54 degree rotation in 2D with 97% success rate**			
3	7	1.0	572.9
5	7	0.49	219.7
7	7	0.39	141.9
9	7	0.4	124.0
**11**	**7**	**0.4**	**103.9**
13	7	0.3	141.8
11	5	0.29	137.1
11	9	0.4	261.3
11	11	0.35	147.8
**2D simulation B: 36–72 degree rotation in 2D with 97% success rate**			
3	7	1.0	646.3
5	7	0.6	305.5
7	7	0.62	179.7
**9**	**7**	**0.51**	**118.0**
11	7	0.501	158.5
13	7	0.495	168.0
9	2	0.51	317.1
9	5	0.5	150.7
9	11	0.55	145.2
**2D simulation C: 72–108 degree rotation in 2D with 97% success rate**			
3	7	1.0	480.4
5	7	0.449	282.5
7	7	0.255	149.7
**9**	**7**	**0.425**	**138.2**
11	7	1.0	262.37
9	3	0.229	204.8
**3D simulation: 18–36 degree rotation in 3D with 90% success rate**			
3	5	0.5	0.203
**5**	**5**	**0.4**	**0.059**
5	3	0.7	0.089

*The bold rows are the models with best performance for the four different experiments in Section 4*.

As can be seen from the table, a number of models having PCA dimensions from 7 to 13 and number of Gaussians ranging from 5 to 11 are seen to perform fairly well. However, there are some clear winners: the 11-dimensional model for test A and the 9-dimensional model for tests B and C, all with 7 Gaussians. Note that models with very small PCA dimension or very low numbers of Gaussians tend to perform poorly.

### 4.2. 3D Simulation

3D soft hand simulation was also created to test this method. The setup for the 3D simulation is shown in Figure 4 (bottom). The 3D simulation is implemented with the SOFA stdlib library, a simulation library for deformable objects (Coevoet et al., 2017). We design a 3D soft hand geometry shown in Figure 4, which has three cylinder shape fingers being planted on a roof shaped palm. The three fingers are actuated by 9 vertical tendons planted on their surfaces. We set the goal of our 3D hand to be rotating an object to a certain degree with respect to the y axis. Then if our method can capture the parameter space of 3D soft hand's action accurately, it should generate actions that can successfully rotate an object to a fixed range of orientations in a variety of ways.

We record the state of the 3D simulation as a vector ***s***_*t*_ = ***q***_*t*_ at time *t*, where ***q***_*t*_ ∈ ℍ is the quaternion of the rigid object being rotated.

The action command at time step t is ***a***_*t*_ = [*a*_*t*,0_, …, *a*_*t*,8_], where and *a*_*t, i*_ is the constraint length of the *i*th tendon at time step t. The action command in our setup is parameterized by the final actuation of all nine tendons $\theta =\left[\theta_{0},\ldots ,\theta_{8}\right]\in {\mathbb{R}}^{9}$, where θ_*i*_ is the final actuation of the *i*th tendon. We generate the action command sequence by gradually shortening the tendons toward their final length. Assuming that ith tendon begins at an actuation of zero and length of *l*_*i*_, the action at time step t can be rewritten in terms of ***θ*** and ***l*** = [*l*_0_, …, *l*_8_] as $a_{t}=l+\frac{t}{T}\theta$.

To investigate the action space of this simulated 3D soft hand, we model the distribution of parameter ***θ*** with our method. Therefore, if our model accurately captures the behavior of the 3D soft hand, the samples drawn from our model should result in different families of actions successful in achieving the rotation task.

We set up experiments to rotate a long and thin cuboid placed on top of a platform by 30 degree about the y axis. Criteria for success for this simulation were (1) the rotation angle is within the designated range (18–36 degrees), and (2) the rod rotates about an axis with magnitude difference from vertical less than a given threshold (0.2 in our experiment). Figure 5 shows a filmstrip of one successful manipulation.

In this case, 1,170 successful samples were collected during initial sampling, and the final number of successful samples that were collected as a result of all of the model building processes was 10,575. Running times were approximately 4 h for collecting initial samples and 7 h for building, refining, and reducing variance for a single choice of model with a single choice of *m* and *k* on a 3.6 GHz Intel(R) Core(TM) i7-4790 CPU.

Results for models built with different *k* and *m* and 90% success rate are shown in the bottom section of Table 2. Here, the 5-dimensional models performed well, with the model having 5 Gaussians achieving the best results.

### 4.3. Visualization of Processes and Results

To provide insight into the sampling process and results, we present data collected for all four experiments at four stages of our method. Figure 3 records the change of sampled data distribution and size for all four rotation tests we set up above. Each column of the figure shows the data collected by one test, while each row corresponds to five different stages of our method.

To facilitate the visualization of the parameter space, for 2D simulation we always choose to show the same 2D slice of data. We found dimensions 7 and 8 to be an informative slice for the 2D simulations, showing two disconnected modes in all three cases. This slice compares the horizontal endpoint of the left finger to the vertical endpoint of the left finger. The region to the top right is a region where the fingers swap places, while the larger region toward the bottom left contains more conventional manipulations, often with much less finger movement.

The first row of the plot records the initial data we collect from the stratified sampling process. The second row shows additional samples drawn from the relaxed Gaussian sampling stage (Figure 2, second row). The third row shows the samples that have been collected after downsampling and then refining each model (third row in Figure 2). Note how the sparsely populated secondary region (representing swapping locations of the fingers) has filled out in the 2D cases. The fourth row shows 1,000 new samples taken from the final GMM, labeled with their successes and types of failure (100 samples for 3D). The fifth row shows 1,000 new samples drawn after variance reduction (last row of Figure 2), also labeled as successes and types of failure (100 samples for 3D). Note how few failures remain in row 5, because the desired success rate has been achieved through variance reduction.

## 5. Discussion

This section discusses the results presented in section 4. We want to demonstrate first that the dimension reduction of Ω helps us to simplify the object manipulation problem and create models that better fit the space. Afterwards, we continue the analysis of the results by analyzing the visualization of our manipulation control planning strategies sampled from our models in section 4. Eventually, we discuss the method's assumptions and its limitations in solving manipulation problems with higher-degree parameter spaces.

### 5.1. Benefits of Reducing Dimension of Ω

The benefits of dimension reduction are evident in our results of 2D simulation experiments. We compare our best models against the almost full-dimensional model built with parameters *k* = 7, *m* = 13. The best models, which have minimum distance from the test data, always have lower dimension than the 13-dimensional model, in all three 2D tests (Table 2). Reducing the dimension of parameter space Ω gives us better models than those built on the full-dimensional space because it helps us to limit the sampling space in the iterative data refinement process. The lower dimensional space allows us to focus on exploring the variance of Ω in a few major dimensions, while ignoring the variance in these less significant dimensions of the space. On the other hand, working in the full dimensional space requires us to explore variance of all dimensions, distracting us from fully exploring some major directions. Given limited sampling time, eliminating the less significant dimensions in order to refine the model resulted in more complete exploration and coverage.

### 5.2. Advantages and Disadvantages of Multiple Gaussians

We dedicate this section to analyze the visualization of our planning strategies, in other words, the process that the simulated soft hand follows our strategies to rotates a rod, in the Supplementary Videos.

The visualization shows the advantage that different Gaussians of our model can generate different families of motions. For example, for the 72–108 degree counter-clockwise rotation in 2D, our best model has in total 7 Gaussians, 4 of which initiate motions that keep the left finger at its original position and let the right finger push upward, another Gaussian makes the two fingers exchange their positions and the last two Gaussians force the left finger to leave its original position until the very last moment while letting the right finger to primarily control the rotation of the rod in this process. From the animation, we can tell that there are not only different clusters forming in the parameter space Ω as shown in section 4.3, but also different families of motions shown in the visualization of the strategies sampled from our model in parameter space Ω. The visualization demonstrates the variety of motions could be generated from our planning. Moreover, it demonstrates that different Gaussians of our model can generate different families of motions. Thus, people can use our model to find different manipulation planning strategies and select certain strategies for the hand by picking corresponding Gaussians of our model.

However this “strategy selection by Gaussian” process is slightly inefficient, since each individual Gaussian would not always give rise to motions significantly different from that of another Gaussian. Thus, to determine the Gaussians that are generating similar motions, we need to manually check the animation of their samples. This process could be accelerated by a metric that automatically evaluates the difference between the Gaussians of our model so that it can help us to decide if two Gaussians generate similar strategies or motions. To design this metric, we can test our method in a lot more different simulation and physical context, so that more data about the correlation between soft hand motions and the parameter space could be gathered to help us understand the relationship between these two space. However, this also means that the metric might only be able to work with certain dynamics model being assigned to either the simulation or the physical hand. The environment/dynamics model assumed by the metric would reduce the application scope of our method and defies our approach's model-free nature. Therefore, for now, the manual process is still beneficial since it keeps our method model-free and applicable to a wider range of soft hand planning scenarios.

### 5.3. Assumptions and Limitations

Our assumptions about the problem limit our method to only controllers whose parameter space is continuous. On top of that, our method is incapable of discovering successful parameter ***θ*** that are far from the successful data Θ_*succ*_ collected in the initial full-space sampling process. This is because later steps of constructing the GMM assume that we have already fully explored the boundary of the parameter space in the initial full-space sampling process and proceed to augment the successful data set by filling in the space around existing successful samples. Thus, after we finish the initial full-space sampling process, little variance would be added to the data set later in our method.

Because the coverage of our model is limited to the variance of successful data we discovered in the full-space sampling process, our method would perform less efficiently for very high dimensional parameter spaces. High-dimensional parameter spaces make it harder to hit some successful data in the full-space sampling process, and limit the variance of motions that our final GMM can generate. Therefore, to make our method successful for a high dimensional parameter space, we need to gather more samples from stratified sampling, increasing the running time required for our method.

### 5.4. Future Directions

There are several future directions for us. One is to further validate the robustness of our method by setting up the real world scenario that our simulation tries to emulate. Testing this method on manufactured soft robots will enable us to evaluate the method's effectiveness for real-world applications.

Another direction is testing out more manipulation tasks for our method. In our current experiments, we only test our method's performance in rotating objects. However, there are many other useful tasks could be considered as our manipulation goals, e.g., grasping, throw, and catch. With more manipulation tasks' models being learned, we can combine these unit manipulation tasks into some more complex behavior.

Also, our method's application can be extended to optimizing design of soft robotic hands. The model we constructed can help evaluate the effectiveness of soft robotic hands' design in achieving certain manipulation goals.

**Figure 6:**
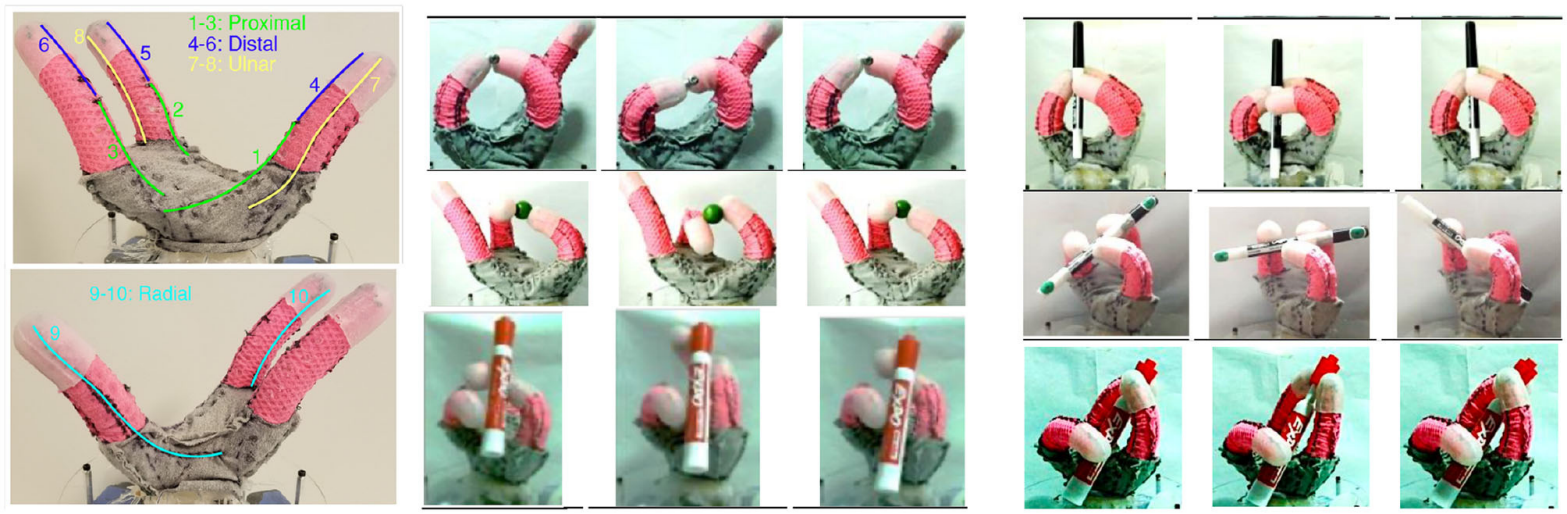
Ten degree-of-freedom dexterous soft foam robot hand developed by Coulson (2020), along with some of manipulations it can perform. The technique described in this paper can be used to identify continuous families of manipulations centered around the actions shown in the future.

One concrete example of future work is inspired by the results shown in Figure 6. The 10 degree-of-freedom soft hand in this figure was specifically designed by Coulson (2020) to accomplish in-hand manipulation tasks. Some examples are shown in the figure. These manipulations were programmed in an open loop manner. Investigation of the entire suite of manipulations showed that the majority could be performed well on the real robot with two keyframes, operating in a reduced-dimensional space of only three dimensions. The set of behavior families corresponding to these manipulations could thus be created using a six-dimensional parameter space Ω and explored in simulation, on the robot, or using a mix of the two modalities, in order to move this collection of pre-programmed capabilities toward a suite of action families that can robustly and flexibly accomplish these manipulations. Apart from the expected value of improving the robustness and flexibility of the interactions that were previously programmed open-loop, competing hand designs can be compared and the hand refined based on the resulting volumes of action spaces generated.

We look forward to continuing this line of research and building on these efforts. We believe there is substantial value in attempting to represent a global space of hand capabilities and look forward to creating such representations for soft robot hands such as those represented in this paper. We note, however, that the approach is fully general and can be applied to any robot where it is possible to sample and evaluate an action space.

## Data Availability Statement

The raw data supporting the conclusions of this article will be made available by the authors, without undue reservation.

## Author Contributions

JS was responsible for developing the research idea and for producing the majority of the results. JK was responsible for supervising the aspects of the research related to the use of soft robotic hands, as well as for assisting with data analysis. NP was the faculty advisor for the research project. All authors contributed to the article and approved the submitted version.

## Conflict of Interest

The authors declare that the research was conducted in the absence of any commercial or financial relationships that could be construed as a potential conflict of interest.

## Supplementary Material

A video supplement demonstrating the results can be found at: https://youtu.be/ZwRDwymXH6Q.
